# Concealed Placental Abruption Complicating Hypertensive Disorders of Pregnancy: Exploring the Role of Point-of-Care Ultrasound

**DOI:** 10.3390/diagnostics16030478

**Published:** 2026-02-04

**Authors:** Michele Orsi, Dereje Merga, Firanbon Negera, Wasihun Shifata, Ashenafi Atomsa, Flavio Bobbio, Admasu Taye

**Affiliations:** 1 Fondazione IRCCS Ca’ Granda Ospedale Maggiore Policlinico, 20122 Milan, Italy; 2 Saint Luke Catholic Hospital, Wolisso P.O. Box 250, Ethiopia; 3 Doctors with Africa CUAMM, 35121 Padua, Italy

**Keywords:** placental abruption, hypertensive disorders of pregnancy, point-of-care testing, obstetric ultrasonography, pregnancy

## Abstract

Placental abruption (PA) without vaginal bleeding is known to be associated with severe outcomes when compared to symptomatic cases; the presence of hypertensive disorders of pregnancy (HDP) is an additional negative prognostic factor. According to guidelines, severe HDP are indications for prompt delivery after maternal–fetal stabilization. Considering gestational age, parity and clinical obstetric examination, the induction of labor should be prioritized to avoid additional risks associated with cesarean section. However, since only a minority of cases of PA may be detected by ultrasonography (US), findings consistent with this suspicion should contribute to the establishment of an appropriate mode of delivery. We present two cases affected by severe HDP, eclampsia and HELLP syndrome, admitted to St. Luke Catholic Hospital, Wolisso, Ethiopia. In both cases, obstetric point-of-care (POC) US revealed a live premature fetus and a solid heterogeneous placental mass, raising the suspicion of concealed placental abruption. To expedite delivery, cesarean section was promptly offered. PA was confirmed in both cases; the first had stillbirth and postpartum hemorrhage, while the second ended up with healthy mother and newborn. In conclusion, POC-US imaging could play a role in optimizing delivery mode and timing for patients with HDP in low-resourced settings. Additional research is warranted to determine the impact of this technique in the management of obstetric emergencies.

**Figure 1:**
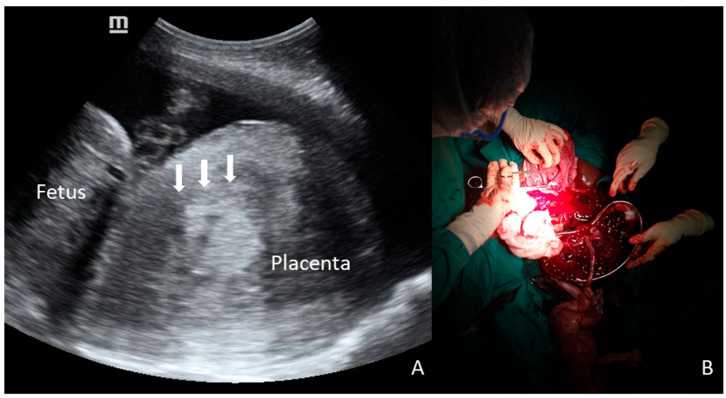
A 28-year-old primigravid woman at 29 weeks of gestation was admitted with antepartum eclampsia. Fetal heart rate was regular upon admission, but POC-US (**A**) showed an unusual shape of the placenta, represented by a voluminous, round, inhomogeneous, and Doppler-negative mass, raising suspicion of concealed PA (arrows). Despite the proposal for emergency CS, the patient initially refused surgery, and extensive counseling with her family was required. During this period, fetal surveillance was intermittent due to the unavailability of continuous monitoring. After a delay of several hours, CS was performed, and a massive PA was confirmed; the final outcomes were stillbirth and obstetric hemorrhage (800 mL of retroplacental clots, **B**).

**Figure 2:**
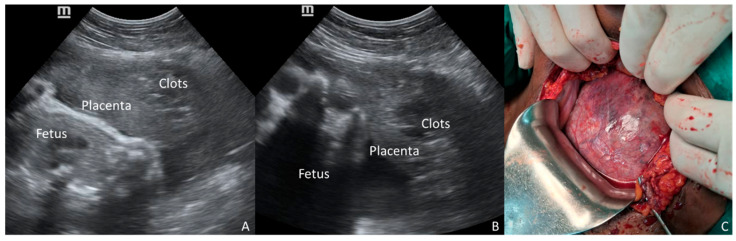
A 32-year-old multigravid woman at 33 weeks of gestation, who was admitted for preeclampsia, experienced a sudden onset of severe hypertension associated with epigastric pain. POC-US was promptly performed along with lab investigation and stabilizing therapy. While the anterior placenta was normal, a dishomogeneous and hyper-hypoechoic mass was noticed between the placental tissue and the uterine wall (**A**,**B**). High suspicion of partial concealed abruption was raised. Emergency CS was performed without delay. The diagnosis of massive PA was confirmed at surgery, and a Couvelaire uterus was demonstrated (**C**). Lab tests concurrently confirmed HELLP syndrome. Placental abruption (PA) is a devastating obstetric emergency complicating approximately 1% of pregnancies and remains a leading cause of perinatal mortality and severe maternal morbidity [1,2]. Given that the diagnosis of PA is primarily clinical and traditional diagnostic tools like ultrasonography (US) and Doppler are notably limited in their predictive value [1,2], classifying the severity of PA has become critical. Two-thirds of PA cases are defined as severe, carrying a distinctively higher morbidity risk profile compared to mild abruption or non-abruption cases [3]. The diagnosis is complicated in cases of concealed placental abruption—where external hemorrhage is absent—or when the primary symptom is abdominal pain. These presentations are consistently associated with significantly poorer outcomes, including higher rates of intrauterine fetal death (IUFD) and severe maternal complications like disseminated intravascular coagulation (DIC) and uteroplacental apoplexy [4,5]. Indeed, in a significant minority of cases, neither vaginal bleeding nor pain is present, rendering the diagnosis dependent solely on non-specific systemic signs [6]. The presence of hypertensive disorders of pregnancy (HDP)—including severe preeclampsia, eclampsia, and *Hemolysis-Elevated Liver enzymes-Low Platelets* (HELLP) syndrome—acts as a significant, compounding risk factor for PA. The co-occurrence of PA and HDP is associated with poorer maternal and neonatal outcomes, including higher rates of severe abruption and stillbirth, compared to PA occurring in normotensive women [7,8,9]. Furthermore, in patients with preeclampsia, symptoms often shift away from typical vaginal bleeding toward non-specific central nervous system symptoms [10], masking the underlying abruption. Standard management for severe HDP dictates prompt delivery after maternal stabilization. The decision between induction of labor (IOL) versus immediate CS is critical. While IOL may be preferred, the presence of a massive, concealed PA makes CS mandatory to expedite delivery and prevent catastrophic hemorrhage. Traditional US has notoriously low sensitivity for diagnosing PA, with retroplacental hematoma seen in only a minority of cases (as low as 15%) [6]. However, given the extreme risk associated with occult PA in the setting of severe HDP, any suspicious finding demands attention. Point-of-Care Ultrasound (POC-US), a valuable tool for rapid, bedside evaluation of critically ill patients [11,12], offers a unique opportunity for quick, targeted diagnostic imaging to assess the placental interface. We presented two illustrative cases of severe HDP—one with eclampsia and one with HELLP syndrome—managed at St. Luke Catholic Hospital in Wolisso, Ethiopia. In both instances, POC-US revealed a retroplacental mass, which raised the suspicion of concealed PA and influenced the decision-making process toward immediate surgical delivery. These cases underscore the potential of POC-US findings to act as a contributing triage tool for obstetric emergencies occurring in a low-resourced setting. The two clinical cases presented here vividly illustrate the critical diagnostic and therapeutic challenges posed by concealed PA when complicating severe HDP. In both patients, the suspicion of PA was raised by a retroplacental mass visualized via POC-US, leading to the choice of immediate CS. Crucially, both patients lacked the classic symptoms of PA (vaginal bleeding or severe abdominal pain), placing them into the high-risk “concealed” category. Although several conditions may increase the risk of PA—i.e., advanced maternal age, trauma, amniotic fluid abnormalities, cocaine use—the literature consistently highlights the severe synergistic effect of HDP, compounded by the concealed presentation [1,7,9]. Studies focusing on symptom types demonstrate that when PA is concealed or presents primarily with abdominal pain, maternal and neonatal outcomes are dramatically poorer compared to revealed cases (vaginal bleeding) [4,5]. Although POC-US cannot be promoted as a primary screening tool for placental abruption due to its limited sensitivity, we emphasize its role as a multipurpose triage tool. The 2026 ISUOG Practice Guidelines (Recommendation 2) suggest POC-US for placental localization in cases of antepartum hemorrhage [12]. However, as demonstrated in our cases, POC-US performed for standard indications—such as assessing fetal viability or gestational age—can incidentally reveal retroplacental masses even in the absence of overt bleeding. It is well-established that ultrasonography (US) is neither sensitive nor specific for the diagnosis of PA, with detection rates ranging from 25% to 60% [13]. The lack of robust specificity data means that a positive US result does not definitively confirm PA. However, in the critical context of severe HDP combined with the high fatality rates of concealed PA, the objective evidence of a retroplacental mass, even if only raising suspicion, may dramatically shift the risk–benefit analysis toward immediate surgical delivery to prioritize maternal and perinatal safety. Our first case demonstrates the risk of delayed intervention; despite the POC-US finding of a retroplacental mass, a surgical delay resulted in intrauterine fetal demise (IUFD) and severe maternal morbidity, namely obstetric hemorrhage. This tragic outcome suggests considering a positive POC-US finding in this high-risk scenario as an indicator of surgical emergency. In contrast, the second case highlights the optimizing role of POC-US; the finding of the placental mass provided objective evidence to bypass potential induction and proceed directly to immediate CS, contributing to a favorable maternal and neonatal outcome despite the massive PA found intraoperatively. In both cases, the US examination was performed by Integrated Emergency Surgical Officers, who are non-medical providers trained according to a specific national program that ensures competence in obstetric POC-US. The utility of this technology is amplified in low-resource settings, where access to specialized diagnostic services or continuous cardiotocographic monitoring (CTG) is typically limited [11,12,14,15]. A rapid, bedside POC-US scan, confirmed to be reliable in similar environments, becomes a life-saving triage tool [11,12,14,15]. It may support the clinical team to differentiate a severe HDP requiring medical stabilization and labor induction from one complicated by an acute, catastrophic event—concealed PA—that requires immediate operative delivery. The objective visualization of suggested findings provides their necessary confirmation in an environment where clinical judgment must be promptly offered but is often based on minimal resources. Nonetheless, the technical limitations of POC-US must be recognized; it remains highly operator-dependent and carries a risk of false-negative results, particularly in cases of posterior placentation where fetal shadowing may obscure the retroplacental interface. In conclusion, this report demonstrates that a positive POC-US finding consistent with retroplacental hemorrhage in the setting of severe HDP—eclampsia or HELLP syndrome—should be viewed as a critically significant indicator of concealed PA, suggesting a shift toward immediate CS. The reliability of POC-US devices in low-resource settings, combined with their speed and accessibility, makes them a powerful tool for optimizing emergency obstetric management. Additional prospective research is warranted to accurately determine the positive and negative predictive value of POC-US in the high-risk obstetric population.

## Data Availability

The original contributions presented in this study are included in the article. Further inquiries can be directed to the corresponding author.
